# The Impact of Group Size on Welfare Indicators of Ewes during Pregnancy

**DOI:** 10.1371/journal.pone.0167061

**Published:** 2016-11-28

**Authors:** Xavier Averós, Ignacia Beltrán de Heredia, Roberto Ruiz, Inma Estevez

**Affiliations:** 1 Department of Animal Production, Neiker-Tecnalia, Vitoria-Gasteiz, Spain; 2 IKERBASQUE, Basque Foundation for Science, Bilbao, Spain; University of British Columbia, CANADA

## Abstract

Group size (GS) and space allowance have major implications for the welfare of production species, however their effects are often confounded. In a previous study we investigated the impact of varying space allowance at constant GS. In the present work we report the consequences of varying GS on pregnant ewes while controlling space allowance. We housed ewes at 6 (GS6) or 12 ewes/enclosure (GS12), while controlling space allowance to 1.5 m^2^/ewe (3 enclosures/treatment), and necessarily varying enclosure size. Therefore, when indicating GS effects we implicitly reflect a confounding effect with that of enclosure size. Movement, use of space, behaviour, serum cortisol concentration and body condition score (BCS) were collected during the last 12 gestation weeks. Movement, use of space, and behaviour were collected every other week, during 2 days/week, using 10 minute continuous scan samplings. Blood was collected during weeks 10, 13, 17, and 21 of gestation, and BCS during weeks 15 and 21. Data were analysed using repeated measures, generalized linear mixed models, with GS, week, and their interaction as fixed effects, and enclosure as random effect. GS mainly affected movement and use of space. GS12 ewes walked longer distances using longer steps (P<0.001). An interaction GS by week was observed for angular dispersion (P<0.0001), which was smaller for GS12 from week 10 onwards. Initial restlessness levels were lower for GS12, as shown by the reduced frequency of location changes (P<0.0001). Furthest and mean neighbour distances increased with GS (P<0.0001). The effect of GS on behaviour was only evident for eating behaviour as an interaction with gestation week (P<0.05). Changes in behaviour, movement and use of space along the study indicated an activity peak during weeks 3 to 5. Cortisol changes during gestation (P<0.01) also reflected this activity peak, while BCS (P<0.001) reflected normal physical condition changes during pregnancy. Although the separate effects of GS and enclosure size cannot be disentangled, we conclude that if enough space/ewe is given during gestation, larger GS will result in larger effective space, and no major implications for the welfare of ewes should be expected as GS increases. Ewes will adapt their movement patterns and use of space to enclosure size, and no further behavioural, physiological and physical consequences should be expected.

## Introduction

Most animal species form social groups, which represents a trade-off between the costs and opportunities of belonging to a community [1]. In the wild, an optimal group size (GS) is the resulting balance between benefits such as higher probability of survival to predation and species perpetuation, and drawbacks such as higher competition for access to resources when these are scarce [2]. Continuous re-adjustments are therefore required to maintain a reasonable social stability, and wild animals can join or leave a community if necessary. This is not an option for farm animals, which are usually reared within static groups in which any conflict cannot be solved by GS readjustments. This impossibility of self-regulation may, in some instances, lead to a deterioration of the welfare status of the group, which highlights the importance of GS for farmed animals [3].

Under production conditions GS is established by farmers, considering aspects mostly related to labour saving and economic profit, with the animal perspective being often overlooked. Large GS may be advantageous for farm animals, since increased social tolerance will lead to a reduction of aggressions [4], but they may become inadequate if resources are limited, such as the case of high stocking densities or limited access to feed [4]. In such cases larger GS may be stressful and, in severe cases, lead to reduced welfare and performance.

Dairy sheep have traditionally been reared under extensive conditions characterized, among others, by access to extensive grazing areas [5] where flocks have higher opportunities to adjust GS, form subgroups or leave according to the availability of local resources. Nonetheless, the adoption of more intensive husbandry practices, with the aim to minimize the negative effects of feed shortage and adverse climatic conditions, has resulted in sheep being kept indoors during some periods of the year [6]. Dairy ewes are often indoors during part of their gestation, and it is possible that the resulting space restriction and imposed GS have undesired consequences. In this sense, it has been shown that limiting space availability influences ewes’ spatial distribution, movements and behaviour [7,8], even in cases of moderate spatial limitation [9]. Besides space allowance, the effects of GS need also consideration in indoor housed pregnant ewes since it will largely determine their social dynamics [10,11]. A recent study comparing groups of 2 and 10 lambs (at a constant density of 2.4 m^2^/lamb) showed that animals in the largest groups were less active and had slower growth [12]. Comparing groups of 9 and 36 pregnant ewes at 1.5 m^2^/ewe [13], ewes in the largest groups showed lower levels of synchronization and higher variability in their behaviour, although levels of aggression were similar. Lactating ewes housed individually or in groups of 5 and 10 also exhibited different activity levels when subjected to fear tests [14]. Altogether, these studies suggest that GS effects might translate into different levels of stress in indoor housed pregnant ewes. However, it must be acknowledged that in most studies a confounding effect exists between the effects of space allowance, GS, and enclosure size as it has been extensively studied in chickens [15,16]. In this sense, if one of the three effects is kept constant, the other two will unavoidably be confounded. Thus, to investigate the potential effects of GS while maintaining constant the space allowance per individual, enclosure size will have to change proportionally to the number of individuals, resulting in confounding effects of group and enclosure size.

In addition to behavioural changes, potential stress associated to differences in GS would be expected to trigger the activation of the HPA axis, therefore increasing the concentration of blood circulating glucocorticoids [17]. This might result, in severe cases, in the reallocation of body resources to cope with stress, which might be detrimental to their physical status [18]. In this sense, the body condition score has also been suggested to be a valid welfare indicator [19].

In a previous study we investigated the impact of space allowance on the behaviour and use of space of pregnant ewes. In the current study we aimed at determining the impact of GS while maintaining a constant space allowance. It may be anticipated that, if space per ewe remains constant, any potential effect of GS will vary as gestation progresses. In this study we investigated the effect of 2 GS (6 and 12 animals), while controlling for space allowance, on the welfare of pregnant ewes during the last two thirds of gestation. Welfare assessment was carried through a combination of different behavioural, movement patterns, use of space, physiological and physical indicators.

## Materials and Methods

The experiment was approved by the NEIKER-Tecnalia Animal Experimentation Committee (Reference AFA_2011_02), and carried out following the European Directive 86/609/ECC regarding the protection of animals used for experimental and other scientific purposes. The study was designed to detect differences in the behaviour, movement patterns, use of space, serum cortisol concentration, and body condition score of pregnant ewes subjected to 2 GS treatments. Ewes were monitored throughout the whole experiment, and no major health and welfare issues, directly caused by experimental treatments, were observed.

### Facilities and Experimental Animals

The study was conducted at the experimental dairy sheep farm of Neiker-Tecnalia (Arkaute, Spain) between August 2012 and January 2013. One hundred and fifty primiparous and multiparous ewes (*Ovis aries*) of the Latxa breed were subjected to artificial insemination (AI) at the end of August, remaining together afterwards. Ewes were managed as a single flock previous to the experiment and were initially housed in a barn (14 m × 32 m) with solid walls and windows allowing natural lighting. Straw bedding was provided, and fresh straw was periodically added to maintain adequate bedding conditions. Ewes had daily access (about 20 h/day) to an adjacent outdoor pasture (5 ha) until housed in the experimental enclosures. Pasture was complemented with ad libitum access to vetch and oats inside the barn, and water was available through automatic nipple drinkers both at the pasture and inside the barn.

### Experimental Design

Fifty-three days after AI, positive gestation and number of viable foetuses were determined via ultra-sound methodologies (Ovi-scan 6, BCF, Australia). Ewes were also weighed, and their body condition (BCS) was assessed using a 5-point scoring scale [20] by one experienced observer with more than 20 years of experience with the selection of this particular experimental flock. Fifty-four 1 to 5 year old ewes, among those confirmed pregnant, were randomly assigned to 1 of 6 experimental groups corresponding to 2 GS treatments (6 and 12 ewes, GS6 and GS12 respectively), with 3 replicates per treatment. Groups were balanced for initial BCS, age and number of viable foetuses. Space allowance remained constant at 1.5 m^2^/ewe in all cases, and thus enclosure dimensions had to vary according to GS, being 270 × 334 cm for the GS6 and 470 × 383 cm for GS12. Enclosure walls were built of PVC to prevent visual contact across groups. Ewes remained in their enclosures until 25 days after the beginning of the lambing period, although the observations finished right before lambing started. All animals were marked on the back for individual recognition (purple spray, Multi-line, Ukal, France). Although we acknowledge that this experimental design confounds the effects of GS with those corresponding to differences in enclosure sizes, unfortunately it was not possible to include any additional treatment due to limitations in animal facilities and in the availability of enough pregnant females in our experimental flock. We are, however, well aware of the problem and the separation of the effects of density, GS and enclosure size have been thoroughly investigated in the case of chickens [15,16].

Feed was provided in an automatic feeding line, with 8 and 14 individual feeding spaces (48 cm/ewe) for GS6 and GS12 respectively, allowing simultaneous access to feed to all ewes. From gestation week 10 to gestation week 15 ewes were fed silage twice/day, at 08:30 and at 15:00 (about 1.5 kg per ewe and day), complemented with 400–500 g/ewe of a barley and wheat mix during the morning meal, and with *ad libitum* access to oat hay and peas during the afternoon meal. From gestation weeks 15 to 19 silage was provided during the morning meal, while fescue hay was provided during the afternoon meal. The total amount of silage and fescue hay was about 1.5 kg/ewe and day. The diet was also complemented with 400–500 g/ewe of a barley and wheat mix during the morning meal, and with *ad libitum* access to oat hay and peas during the afternoon meal. During gestation weeks 20 and 21 silage and fescue hay were provided as described, but each meal was complemented with 500 g/ewe of concentrate (1.101 UFL/kg; 168 g PB/kg). From gestation weeks 10 to 17 ewes also had free access to salt blocks (TIMAC SAS, St Malo, France). Blocks were substituted by a cube containing vitamin-mineral corrector (INAFORM, Timac Agro, Orcoyen, Spain) afterwards. Drinking water was available *ad libitum* through an automatic drinking nipple installed in each enclosure. Fresh straw bedding was initially provided to ewes in each enclosure, with fresh straw being periodically added to maintain a good bedding condition.

### Behaviour, Movement and Use of Space

Data collection started during gestation week 10 (64 days after AI, after groups were formed) and lasted for 11 weeks (end of gestation week 20, right before lambing). Live observations were carried out by a single observer every other week. To precisely locate ewes during observations, enclosures were divided into a visual grid (32 and 49 squares for GS6 and GS12, respectively) using an alpha-numerical code written on stickers, which were placed along walls according to enclosure dimensions, as previously described [21].

Observations were carried out in the morning, during 2 days/week, starting at 09:30 after the morning meal and finishing before the afternoon meal. Two rounds of observations of all enclosures were conducted during each observation day. During each round enclosures were observed consecutively and each enclosure was observed for 10 minutes (total of 240 minutes/week of observations), with 10 scan samplings/enclosure being collected (1 scan/minute). The order of enclosure observations was random for each round. Randomization was carried out by permutations of experimental enclosures, permutation of enclosures and ewes within each enclosure (www.random.org), or simple random sampling techniques (www.randomizer.org) depending on specific needs. Within each scan the withers position (in XY coordinates; expressed as X and Y distances from the origin of coordinates, in cm) of all ewes in the enclosure was sequentially collected, with the help of a visual grid, using the Chickitizer software [8,22]. The behaviour of each ewe was simultaneously collected with the software following the ethogram shown in Table 1. One ewe from the GS12 treatment died of Listeriosis during the last week of this experiment. Replacing the ewe would disrupt enclosure’s social dynamics and be stressful to animals. Given that the experiment was ending, and no major impact was expected on results, the dead ewe was not replaced and data collection continued regularly.

**Table 1:** Ethogram used during observations.

Behaviour		Definition
Eat		Standing by the feeder, with the head completely inside one of the feeder holes
Explore enclosure		Nose interaction with the enclosure wall of other physical issue
Forage		Standing, with the head down, interacting with the floor bedding with the mouth
Move		Change position within the enclosure, either walking or running
Negative social interaction	Butt	Sudden, strong head contact with another ewe
	Displace from resources	Force another ewe to leave the feeder, drinker, or the mineral resource
	Push	Press the head against another ewe to force the pass
	Threaten	Directing the forehead towards another ewe with no physical contact
Positive social interaction	Sniff	Smell another ewe without physical contact
	Nose	Slightly contact another ewe with the nose
	Groom	Clean the wool of another ewe using the mouth
	Nudge	Slightly, gently push another ewe
	Lick	Lick any part of another ewe’s body
Rest		Lie down on the floor, either ruminating or not
Self-groom		Groom, either by self-licking or by rubbing against a physical enclosure object
Stand, static		Stand with the four feet on the floor, either ruminating or not

During data collection, clicking on an *a priori* identical position will unavoidably result in slightly different XY values. Although the impact in movement and use of space parameters would have been minor, it could result in overestimation of the activity rate. This potential error was corrected considering the enclosure dimensions of each experimental treatment, as previously described [8]. From the corrected XY locations, a series of parameters characterizing ewes’ movement and use of space were calculated [21,23,24]. These included total and net distances, net to total distance ratio, longest and shortest step lengths, furthest neighbour distance, nearest neighbour distance, mean neighbour distance, angular dispersion, and movement activity. Further details on these variables and their definitions are given in Table 2.

**Table 2:** Movement and use of space variables.

Variable	Definition
Total Distance (cm)	Total distance = $\sum_{i=1}^{k}\sqrt{\left({\left(x_{i+1}-x_{i}\right)}^{2}+{\left(y_{i+1}-y_{i}\right)}^{2}\right)}$, that is, the sum of the euclidean distances between the k consecutive locations composing the trajectory of one ewe during one observation period.
Net Distance (cm)	Net distance = $\sqrt{\left({\left(x_{k}-x_{1}\right)}^{2}+{\left(y_{k}-y_{1}\right)}^{2}\right)}$, that is, the euclidean distance between first and last location of the trajectory of one ewe during one observation period.
Net to Total distance ratio	Ratio between Net to Total distances.
Longest step length (cm)^1^	Longest Euclidean distance between two consecutive locations composing the trajectory of one ewe during one observation period.
Shortest step length (cm)^1^	Shortest Euclidean distance between two consecutive locations composing the trajectory of one ewe during one observation period.
Furthest neighbour distance (cm)	Within the same scan, Euclidean distance between the location of a given ewe and that of the furthest ewe within the enclosure.
Nearest neighbour distance (cm)	Within the same scan, Euclidean distance between the location of a given ewe and that of the closest ewe within the enclosure.
Mean neighbour distance (cm)	Within the same scan, mean Euclidean distance between the locations of all the ewes within an enclosure.
Angular dispersion	For the k consecutive locations composing the trajectory of one ewe during one observation period, angular dispersion = $\sqrt{{\bar{x}}_{k}^{2}+{\bar{y}}_{k}^{2}}$, where ${\bar{x}}_{k}=1/k\sum_{i=1}^{k}\cos(\theta_{i})$ and ${\bar{y}}_{k}=1/k\sum_{i=1}^{k}\sin(\theta_{i})$, being θi the turning angle between the ith location and the (i-1)th location.
Movement activity	Frequency of scans in which the position of a ewe differed from that in the previous scan.

^1^: Not to be confounded with a real step taken by the ewe to move that distance between 2 consecutive locations.

### Serum Cortisol Concentration and BCS

Fifty-six days after AI 4 ewes/enclosure were randomly selected for the determination of pre-experiment serum cortisol concentration through 10 ml blood collection. Blood samples from these ewes were also collected during gestation weeks 10, 13, 17, and 21. Blood sampling started at 11:30 in the morning, with the enclosure order, and ewes within each enclosure order, being random. Samples were collected into non-coated, evacuated tubes (Vacutainer® Rapid Serum Tubes, BD, New Jersey, USA), through jugular venepuncture for determination of pre-experiment serum cortisol concentration. Blood sampling was carried out within the enclosure, with the whole sampling process taking about 1 minute/ewe (that is, no more than 4 minutes/enclosure in total), therefore minimizing any potential effect of handling. Blood samples were stored on ice during collection, and were processed immediately after. They were centrifuged at 4°C during 15 minutes at 4000 rpm/2486 *g*. Resulting serum was stored at -20°C until processing. The determination of pre-experimental and experimental serum cortisol concentrations was carried out, on duplicate samples, using a double antibody, enzyme immunoassay [25,26] and spectrophotometric measures. The limit detection was 1 ng/ml, and the intra- and inter-assay CV’s were 8.3 and 12.8% respectively. Additionally, to detect the effect of GS on the variation of the physical condition along the experiment, BCS of all ewes was collected, as previously described and by the same experienced observer, during gestation weeks 15 and 21.

### Statistical Analysis

Mean values of all variables per enclosure and gestation week were calculated. Normality and variance homoscedasticity of data were confirmed and, accordingly, movement and use of space variables were analysed considering a Gaussian distribution. Frequencies of behaviours were analysed considering a binomial distribution except for negative social behaviours in which case, given their low occurrence, a Poisson distribution was chosen. Serum cortisol concentration and BCS were analysed using a lognormal distribution. The effects of GS (DF = 1), gestation week (DF = 6 for movement, use of space, and behaviour variables; DF = 3 for serum cortisol concentration; DF = 1 for BCS), and their interaction on all variables were estimated using a repeated measures, mixed model ANOVA. In the models gestation week was included as the repeated measures unit, and enclosure within GS was included as a random effect. In the models for cortisol and BCS the pre-experiment values were included as covariates. A first order autoregressive covariance structure was assumed for all variables to account for any linear dependence of measured variables over time, except for BCS in which case a variance component structure was assumed. Least square means were computed in case of statistically significant effects (*P*<0.05), with *P*-values adjusted for multiple comparisons using Tukey range tests. All analyses were performed using the GLIMMIX procedure in SAS 9.3 (SAS Institute, Cary, NC, USA).

## Results

### Movement and Use of Space

The effects of GS on movement and use of space are shown in Table 3. Total and net distances walked by GS6 ewes were significantly shorter than those of GS12 ewes, while the net to total distance ratio remained unaffected by GS. Similarly, longest and shortest step lengths, and furthest, nearest, and mean neighbour distances were shortest for GS6 ewes, although for nearest neighbour distance only showed a trend to statistical significance.

**Table 3:** Effect of group/enclosure size (GS) on the movement, use of space and behaviour of gestating ewes.

Variables		GS6		GS12			
Movement and use of space		Mean	SE	Mean	SE	F_1, 24_	*P*-value
	Total distance (cm)	288.4b	38.5	544.1a	38.5	22.06	< 0.0001
	Net distance (cm)	84.7b	8.7	144.2a	8.7	23.36	< 0.0001
	Net/total distance	0.31	0.01	0.29	0.01	0.61	0.4406
	Longest step length (cm)	97.1b	9.3	188.7a	9.3	48.33	< 0.0001
	Shortest step length (cm)	1.7b	0.7	4.2a	0.7	6.12	0.0208
	Furthest neighbour distance (cm)	223.7b	5.6	381.7a	5.6	392.24	<0.0001
	Nearest neighbour distance (cm)	78.8b	1.9	83.5a	1.9	3.02	0.0951
	Mean neighbour distance (cm)	150.2b	4.1	231.7a	4.1	198.37	< 0.0001
	Angular dispersion	0.55a	0.01	0.52b	0.01	14.87	0.0025
	Movement activity (%)	81.4	0.6	83.0	0.6	3.52	0.0729
Behaviour							
	Eat (% of observations)	0.5	15.5	0.4	5.1	0.00	0.9925
	Explore enclosure (% of observations)	1.3	0.4	0.9	0.2	0.57	0.4563
	Forage (% of observations)	4.1	1.6	4.8	0.8	0.14	0.7109
	Negative social interactions (number of observations)	0.2	41.4	0.3	67.4	0.00	0.9991
	Positive social interactions (% of observations)	0.5	0.2	0.6	0.2	0.02	0.8865
	Rest (% of observations)	36.4	10.6	44.3	10.8	0.27	0.6080
	Self-groom (% of observations)	1.2	0.2	1.2	0.1	0.02	0.8946
	Stand (% of observations)	38.2	7.7	34.1	7.1	0.15	0.6982

Within each row, different letters (a, b) indicate statistically significant differences (*P* < 0.05)

**Fig 1:**
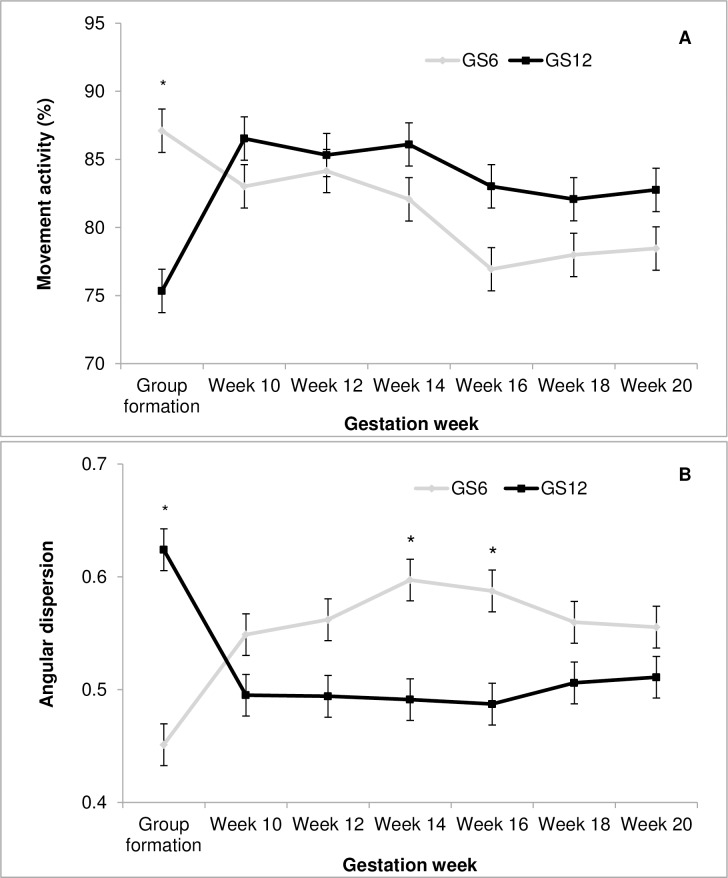
Interaction between group size (GS) and gestation week on the movement activity (A) and the angular dispersion (B) of gestating ewes. Within each week, (*) indicates significant differences between GS (*P* < 0.05).

Movement activity (*F*_6, 24_ = 8.05; *P*<0.0001; Fig 1A) and angular dispersion (*F*_6, 24_ = 14.87; *P*<0.0001; Fig 1B) were affected by the interaction between GS and gestation week. Higher movement activity was detected for GS6 ewes as compared to GS12 at the onset of the study, while angular dispersion was initially higher for GS12, although for weeks 14 and 16 was higher for GS6. No other GS by gestation week interaction was significant regarding the movement parameters.

Regarding changes observed through gestation, total and net distances, and net to total distance ratio were shorter after group formation as compared to weeks 12 and 14, with distances decreasing afterwards for total and net distances (Table 4). Similarly, longest step length was shortest after group formation as compared to gestation weeks 12 and 14. Distance to the nearest, mean, and furthest neighbour were significantly shorter after group formation, followed by a tendency to increase afterwards (Table 4).

**Table 4:** Changes in the movement, use of space, and behaviour of gestating ewes during gestation weeks.

**Variables**		Group formation		Week 10		Week 12		Week 14		Week 16		Week 18		Week 20			
Movement and use of space		Mean	SE	Mean	SE	Mean	SE	Mean	SE	Mean	SE	Mean	SE	Mean	SE	F_6, 24_	*P*-value
	Total distance (cm)	215.1c	59.5	407.7abc	59.5	594.6ab	59.5	648.8a	59.5	338.6bc	59.5	347.4bc	59.5	361.2bc	59.5	6.43	0.0004
	Net distance (cm)	82.6c	12.8	113.0abc	12.8	147.7ab	12.8	152.1a	12.8	90.0c	12.8	97.0bc	12.8	118.7abc	12.8	5.18	0.0015
	Net/total distance	0.40a	0.03	0.28ab	0.03	0.27b	0.03	0.25b	0.03	0.27ab	0.03	0.28ab	0.03	0.35ab	0.03	3.70	0.0096
	Longest step length (cm)	91.6c	15.9	141.0abc	15.9	184.1ab	15.9	197.0a	15.9	122.2bc	15.9	124.5bc	15.9	142.0abc	15.9	5.15	0.0016
	Shortest step length (cm)	3.9	1.2	1.7	1.2	5.6	1.2	5.2	1.2	0.9	1.2	2.4	1.2	1.0	1.2	2.86	0.0302
	Furthest neighbour distance (cm)	266.2b	8.2	302.0a	8.2	311.3a	8.2	313.5a	8.2	306.2a	8.2	314.4a	8.2	305.5a	8.2	4.28	0.0045
	Nearest neighbour distance (cm)	71.1b	2.8	78.7ab	2.8	79.5ab	2.8	82.7ab	2.8	85.7a	2.8	86.3a	2.8	83.9a	2.8	3.11	0.0213
	Mean neighbour distance (cm)	167.5b	5.6	191.8ab	5.6	193.5a	5.6	198.2a	5.6	193.9a	5.6	199.3a	5.6	192.7ab	5.6	4.06	0.0060
	Angular dispersion	0.54	0.01	0.52	0.01	0.53	0.01	0.54	0.01	0.54	0.01	0.53	0.01	0.53	0.01	0.34	0.9112
	Movement activity (%)	81.2ab	1.1	84.8a	1.1	84.7a	1.1	84.1ab	1.1	80.0b	1.1	80.0b	1.1	80.6ab	1.1	5.13	0.0016
Behaviour																	
	Eat (% of observations)	0.0ab	0.0	5.3a	1.2	1.6ab	0.6	1.9ab	0.6	1.8ab	0.6	1.3b	0.5	2.8ab	0.8	3.36	0.0150
	Explore enclosure (% of observations)	0.6ab	0.4	1.7ab	0.4	2.1ab	0.5	2.6a	0.5	0.7ab	0.3	0.8ab	0.3	0.6b	0.2	4.12	0.0055
	Forage (% of observations)	21.6a	3.5	4.4b	1.2	10.8ab	1.8	7.3b	1.5	3.6b	1.1	3.2b	1.0	0.3b	0.4	10.33	< 0.0001
	Negative social interactions (number of observations)	0.0	0.4	2.0	0.7	4.3	1.1	3.3	1.0	1.7	0.7	0.0	0.5	1.7	0.7	1.22	0.3297
	Positive social interactions (% of observations)	0.6	0.3	0.7	0.2	0.9	0.3	0.7	0.3	0.3	0.2	0.7	0.2	0.2	0.1	1.63	0.1812
	Rest (% of observations)	50.0ab	12.3	28.5b	8.5	19.2b	7.1	22.1b	7.4	64.7a	9.5	62.1a	9.6	44.0ab	10.0	6.92	0.0002
	Self-groom (% of observations)	0.6	0.3	2.0	0.3	1.7	0.3	0.8	0.2	1.1	0.3	1.4	0.3	1.6	0.3	2.23	0.0751
	Stand (% of observations)	22.3ab	7.7	47.3a	7.5	48.6a	7.5	52.2a	7.5	22.6b	5.9	23.5b	5.9	43.3ab	7.4	5.40	0.0012

Within each row, different letters (a-c) indicate statistically significant differences (*P* < 0.05)

### Behaviour

**Fig 2:**
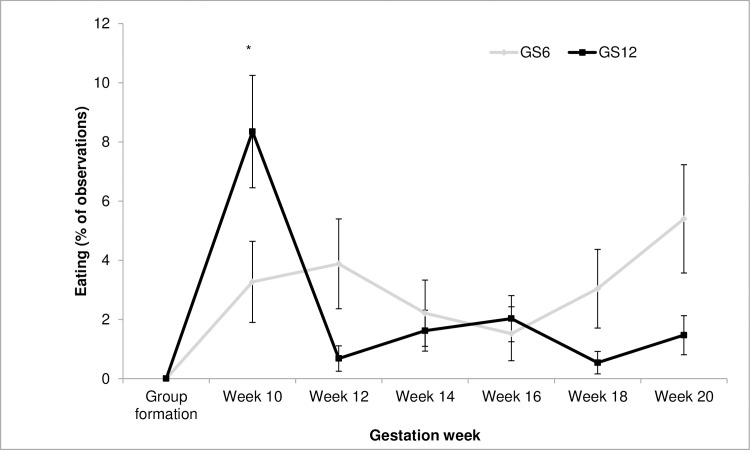
Interaction between group size (GS) and gestation week on the frequency of gestating ewes observed eating. Within each week, (*) indicates significant differences between GS treatments (*P* < 0.05).

Behavioural analysis detected no main effects of GS (Table 3), even for the frequencies of positive and negative social interactions. Only the interaction between GS and gestation week were significant for eating (*F*_6, 24_ = 3.50; *P* = 0.0126) and moving (*F*_6, 24_ = 2.65; *P* = 0.0408). The percentage of ewes eating (Fig 2) during week 10 was significantly higher for GS12 as compared to GS6, while for moving multiple comparisons failed to detect differences between treatment levels.

Somewhat more interesting were the effects of gestation week on behaviour, with the highest enclosure exploration frequencies detected for week 14, and highest foraging frequencies after group formation (Table 4). Both negative and positive social interactions remained unaffected along gestation weeks. The frequency of self-grooming tended to be lowest after group formation and highest during week 10. Initial and final values for resting frequencies were similar, with the lowest values observed for weeks 10 to 14, and the highest during weeks 16 and 18. Standing frequency followed the opposite pattern with highest values detected for weeks 10 to 14 and lowest during weeks 16 and 18.

### BCS and Serum Cortisol Concentration

No effect of GS was detected on BCS (*F*_1,51_ = 0.15; *P* = 0.7004; 2.60 ± 0.11 and 2.67 ± 0.09 mean ± SE for GS6 and GS12, respectively) or cortisol levels (*F*_1,59_ = 1.75; *P* = 0.1907; 5.2 ± 0.7 and 7.6 ± 1.7 for GS6 and GS12 respectively). Changes in serum cortisol concentration were observed as gestation progressed (*F*_3,59_ = 5.12; *P* = 0.0032), with statistically higher cortisol levels detected during gestation weeks 13 and 21 (8.8 ± 3.2 and 8.8 ± 1.7 ng/ml respectively) regarding those observed during week 17 (3.5 ± 0.7 ng/ml), and intermediate values during week 10 (5.0 ± 0.5 ng/ml) that showed no significant differences with the rest of values. Lower BCS was observed in week 21 (2.25 ± 0.07) with respect to week 15 (3.02 ± 0.07; *F*_1,51_ = 89.36; *P* < 0.0001).

## Discussion

The aim of this study was to determine the effect of GS on the welfare of pregnant ewes during the last two thirds of gestation. Two experimental GS treatments, GS6 and GS12, were used while space allowance (1.5 m^2^/ewe) remained constant. Welfare assessment was carried out through a combination of different behavioural, movement patterns, use of space, physiological and physical indicators. It is essential to remark that varying GS while controlling for space allowance unavoidably leads to confusion between group and enclosure sizes, as there is no alternative to control for both space allowance and enclosure size. This problem has been experimentally addressed and discussed in a number of papers [15,16,27]. It is possible to test some of the confounding effects by conducting a more complex experiment, although it is never possible to totally isolate the independent effects of GS, space allowance, and enclosure size [15,16]. In a previous study the effects of density while maintaining a constant GS, but enclosure size changed [7,8]. In this study we tested the effects of GS while maintaining space allowance constant, thus enclosure size changed. Therefore, we must keep in mind that as GS change enclosure size also change, and thus their effects are difficult to discern.

Despite the difficulties in interpretation, the results from our study are relevant as management practices and potential legislation are normally decided according to an established space allowance. In this sense, the results of this study clearly showed that, even when space allowance remains constant, maintaining larger groups in larger enclosures is advantageous from a welfare perspective, as compared to smaller groups in smaller enclosures, as it allows a greater freedom of movement to pregnant ewes, while no negative behavioural incidences were detected. In fact, the effects of the experimental treatments were almost restricted to effects on movement and use of space patterns. Ewes in GS12 walked longer distances, and their movement trajectories were composed of longer steps; neighbour distances were larger, and their trajectories were more tortuous. Group size also influenced the variation in eating frequencies. In a previous study with pregnant ewes, in which GS remained constant while space allowance varied [7,8], major differences in movement, use of space, and social interactions occurred between 1 m^2^/ewe and the other treatments (2 and 3 m^2^/ewe), suggesting the existence of a threshold for the occurrence of behavioural changes between 1 and 2 m^2^/ewe. Space allowance in this study remained constant at 1.5 m^2^/ewe and, interestingly, no treatment effect was observed over the occurrence of positive and negative social interactions, which were lower than that observed in the previous study with ewes housed at 1 m^2^/ewe.

It is generally assumed that, for a given space allowance, the amount of space is the same for all individuals, and does not depend on GS. However in practice, and as previously proposed [28], at constant space allowance animals housed in larger groups, such as GS12, have more effective space as compared to smaller GS6 groups. Not only the total area potentially available for movement is larger, but also the available space is more efficient, resulting in higher movement even though space per individual remained constant. This effect can be detected by the differences obtained in the total and net distances, which were longer for GS12. These differences many have been probably due to the need for longer displacements within the enclosure to gain access to resources such as feed, water, and mineral blocks. On the other hand, angular dispersion was smaller, and therefore trajectory tortuosity was higher [29], for GS12 from week 10 until the end of the study, although differences were actually significant only during weeks 14 and 16. This would be explained by a more prevalent barrier effect in the GS12 treatment in the sense that, when moving around the enclosure, ewes had higher chances to come across other ewes. This barrier effect has been previously described for pregnant ewes [8] and other species [4,30]. Given that effective space permitted it, GS12 ewes would have opted for adapting their trajectories and avoiding contact with enclosure mates rather than interacting with them. These results suggest that, for a given space allowance, changes in quantitative aspects of a trajectory (length) as GS increases appear to be the results of the largest effective space. On the other hand, changes in qualitative aspects (tortuosity) can be explained by a more prevalent barrier effect caused by the presence of a larger number of individuals within the enclosure. On the other hand GS12 ewes kept longer furthest and mean neighbour distances, what would agree with previous results obtained in broilers [21], although in that study the enclosure size was not changed, and so GS was confounded with density, which is not the case in the present study. In poultry, it is known that enclosure size has a stronger effect on the group dispersion than GS [16,31], although stocking density modulates this effect [15]. Given that larger groups were housed in larger enclosures, it is likely that ewes in our study simply tried to maximize space occupation through adjustments in inter-individual distances.

The experimental treatments also had an impact on movement activity and angular dispersion after group formation, with ewes in GS6 changing location more frequently and also described more tortuous trajectories. This suggests a differential response to the novel environment, with initial restlessness levels being higher when GS was smaller. In our previous study (GS kept constant at 6 ewes) an initial adaptation period also occurred, mainly characterized by higher movement activity irrespective of space allowance [8]. Although maybe not strictly comparable, similarities between both experiments for a GS of 6 ewes allow to speculate that behavioural strategies to cope with the novelty might be GS-dependent rather than space allowance dependent. Movement and use of space also indicate that, after this initial period, an activity peak occurred between weeks 12 to 14, and this was independent from GS. The peak was characterised by longer enclosure displacements and longer steps. A relative decrease in the activity levels was observed afterwards, likely attributable to the proximity of the lambing period [7]. Distance to the nearest and furthest neighbour were higher at the end of gestation, and in the case of nearest neighbour distance a gradual increase was observed as gestation progressed, similarly to previous results [8], what would be attributable to the gradual increase in body size. It is also possible that during initial stages of the study ewes might have stayed closer to better cope with the novel situation, but once accustomed to their new environment they gradually appeared to increase their inter-individual distances.

Despite the large differences found in regard to movement patterns, the experimental treatments showed a surprisingly limited effect on the behaviour of the ewes. The interaction between GS and gestation week was significant only for eating behaviour, and would to some extent agree with the reported increase in the percentage of time eating in larger groups (36 ewes vs. 9 ewes) [13]. It might be argued that changes in the diet might have affected ewes, but this would have been the same for all experimental groups. Also, the low frequencies for this behaviour are easily explained by the fact that observations started right after ewes had eaten their morning meal. This interaction, together with results obtained for movement and use of space, suggest that GS12 ewes were initially more active, showing a higher number of changes in location and in associated adaptations of movement trajectories. Results also suggest that the novelty effect was associated to a relatively high frequency of foraging activities, which were highest after group formation and independent of GS. It is therefore possible that initial differences are related to the movement of ewes as they were foraging, and that foraging is actually reflecting exploration of the novel environment.

Negative and positive social interactions were not affected by GS either. This would confirm previous results [13], further supporting the evidence that the increase of effective space associated to increasing GS at constant space allowance does not result in higher levels of social disturbances, which appears to be more closely linked to space allowance [7]. Otherwise, changes in behaviour would be mainly attributed to the progression of gestation, with the lowest resting and the highest standing frequencies being observed during weeks 10 to 14, and with a peak in the exploration frequency during week 14. On the other hand, changes in the resting and standing frequencies during later weeks would be due to the gradual increase of ewes’ body volume and weight, so that they prioritized lying down to standing, what would agree with movement and use of space results, as well as with previous results [7]. The tendency to change in resting and standing frequencies during week 20 might be attributed to discomfort immediately before parturition, with more frequent posture changes rather than displacements around the enclosure.

Whilst it must be accepted that the HPA axis responsiveness is decreased during pregnancy [32], what might have interfered with ewes’ HPA axis activation in response to environmental stimuli, the lack of a treatment effect on serum cortisol levels suggests that the physiological response of pregnant ewes to stress was independent of GS. Considering that body resources are mobilised to cope with persistent sources of stress [18], differences in BCS across treatments should be expected if different GS had resulted in different levels of stress, but this was neither the case. The lack of an effect of the experimental treatments on cortisol and BCS therefore suggests that any potential effect was satisfactorily addressed by ewes through simple movement and use of space adaptations, making the activation of additional physiological, coping mechanisms unnecessary. Increased cortisol levels during week 13 with respect to week 17 might be explained by increased activity levels during the central part of the experiment, as it is known that physical exercise correlates with blood circulating cortisol concentration [33]. Later Increase in cortisol during week 21 could be the result of the necessary physiological preparation for parturition, since basal cortisol levels during late pregnancy are higher than those of non-pregnant ewes [34]. The significant decrease of BCS observed between weeks 15 and 21 would also be due to a loss of body condition during late pregnancy caused by the elevated foetuses requirements and the development of mammary glands [35,36].

In conclusion, the effects of GS on pregnant ewes appeared to be mainly caused by the largest effective space available as GS, and therefore enclosure size, was larger in order to maintain space allowance constant. Effects were majorly restricted to movement and use of space parameters, with GS12 ewes walking longer distances composed of longer steps. They described more sinuous trajectories due to a more prevalent barrier effect caused by the presence of a higher number of individuals in the enclosure. Inter-individual distances increased with GS, indicating adjustments in the group dynamics as enclosure size got larger. Initial restlessness levels were smaller for GS12 ewes, changing less frequently in location and describing less sinuous trajectories after group formation. The absence of treatment differences in the frequency of social interactions, particularly negative social interactions, suggests that increasing the number of individuals in the group does not translate into differences in their welfare status from a social perspective. Otherwise, behaviour changes during the study can be attributed to normal changes caused by the progression of gestation. The lack of an effect of GS on serum cortisol concentrations and BCS indicates that movement and use of space adjustments sufficed and made the activation of further behavioural and physiological coping mechanisms unnecessary. Their changes along the study reflected normal adaptations as gestation progressed.
